# Meat species authentication using portable hyperspectral imaging

**DOI:** 10.3389/fnut.2025.1577642

**Published:** 2025-04-02

**Authors:** Yuewen Yu, Wei Chen, Dongjie Zhao, Hanwen Zhang, Wenliang Chen, Rong Liu, Chenxi Li

**Affiliations:** ^1^State Key Laboratory of Precision Measurement Technology and Instruments, Tianjin University, Tianjin, China; ^2^School of Precision Instruments and Optoelectronics Engineering, Tianjin University, Tianjin, China; ^3^Department of Ophthalmology, Tianjin Eye Hospital, Nankai University Affiliated Eye Hospital, Nankai University, Tianjin, China; ^4^Clinical College of Ophthalmology, Tianjin Medical University, Tianjin, China; ^5^Tianjin Key Laboratory of Ophthalmology and Visual Science, Tianjin Eye Hospital, Tianjin, China

**Keywords:** hyperspectral imaging, meat adulteration, model transfer, discrimination model, adulteration visualization

## Abstract

**Introduction:**

Meat species fraud seriously harms the interests of consumers and causes food safety problems. Hyperspectral imaging is capable of integrating spectral and imaging technology to simultaneously obtain spectral and spatial information, and has been widely applied to detect adulteration and authenticity of meat.

**Methods:**

This study aims to develop a portable hyperspectral imager (HSI) and a discrimination model for meat adulteration detection. The portable push broom HSI was designed with the spectral resolution of 5 nm and spatial resolution of 0.1 mm, and controlled with the Raspberry Pi to meet the requirement of on situ rapid detection. To improve generalization, the model transfer method was also developed to achieve model sharing across instruments, providing a reliable solution for rapid assessment of meat species.

**Results:**

The results demonstrate that the model transfer method can effectively correct the spectral differences due to instrument variation and improve the robustness of the model. The support vector machine (SVM) classifier combined with spectral space transformation (SST) achieved a best accuracy of 94.91%. Additionally, a visualization map was proposed to provide the distribution of meat adulteration, offering valuable insights for fraud detection.

**Conclusion:**

The portable HSI enables on-site analysis, making it an invaluable tool for various industries, including food safety and quality control.

## 1 Introduction

Meat and meat products are an important source of high-quality protein in the human diet, providing essential amino acids, fatty acids, vitamins, and other nutrients (1–3). However, the higher growth rate in meat consumption leads to fraud in processed meat products (4). Meat species fraud typically involves the substitution of one meat species for another, or the presence of animal species that are not labeled (5). Unlabeled meat may contain allergens or pathogens, increasing food safety risks. Additionally, meat adulteration may violate religious culture and harm the interests of consumers. Therefore, effective supervision is very important for ensuring the suitable development of the meat industry, and reliable detection technologies are fundamental technical support for this goal. The most commonly used methods for meat species detection include biochemical, immunological and molecular methods, such as liquid chromatography, polymerase chain reaction, electrophoretic separation and enzyme-linked immunosorbent assay (6–9). However, these methods often require complex sample preparation, such as tissue disruption, target analyte extraction, and purification, and these pretreatment processes are invasive, cumbersome, and time-consuming.

Taking advantage of rapid, non-destructive and low-cost, the spectroscopic methods, including near-infrared (NIR), mid-infrared (MIR), ultraviolet–visible (UV–VIS), and Raman spectroscopy, have been widely used to detect adulteration and authenticity of meat (10–13). However, most spectral systems using fiber optic probes or integrating spheres only collect reflected or transmitted light in small areas of the sample, which can result in unrepresentative spectral sampling and low accuracy for detecting adulteration in meat with heterogeneous characteristics.

Hyperspectral imaging technology, which collects both spectral and spatial information from a sample, has become a powerful tool for meat authenticity (14). The hyperspectral data cube could be used to characterize complex and inhomogeneous meat samples, as well as identify surface and sub-surface components. Masithoh et al. (15) employed a short-wavelength HSI and partial least squares regression (PLSR) to predict pork content in ground beef, achieving a coefficient of determination ( $R^{2}$ ) of 0.97 and a root mean square error (RMSE) ranging from 2.47 to 2.55%. Zhang et al. (16) combined hyperspectral images with recurrence plot transformation and convolutional neural network (RP-CNN) to detect adulterated mutton, achieving a classification accuracy of 100%. Jia et al. (17) utilized near-infrared HSI combined with genetic algorithm-backpropagation artificial neural network for identifying substitution fraud in ground beef. The sensitivity and specificity of the model were 100%. The methods described above integrate spectroscopy with machine learning and deep learning techniques to achieve high-precision classification and prediction of meat adulteration. Hyperspectral imaging not only provides rich spectral information but also captures surface features of meat, such as texture and color (18). Image analysis allows for the extraction of texture features, which can reveal subtle differences in meat structure related to species or quality. Wan et al. (19) fused spectral and textural features of hyperspectral images to predict myoglobin content in nitrite-cured mutton, and the optimal model achieved a prediction RMSE of 3% for oxymyoglobin (OxyMb) and 3.2% for methemoglobin (MetMb). Kucha et al. (20) obtained mean spectral features, gabor filter features, and wild line detector features from hyperspectral images of pork loin and developed a PLSR model to assess the intramuscular fat quality. These results indicate that the combination of spectral and texture features facilitates the rapid assessment of meat quality, offering a robust tool for adulteration detection and quality control.

With breakthroughs in micro-mechanical systems, micro-dispersive optics and narrow-band filtering systems, portable and handheld spectrometers have become one of the most commercially available platforms for meat monitoring and quality detection (21). Sigernes et al. (22) designed a lightweight push broom HSI with a dispersive element housing printed by a thermoplastic 3D printer. The device is portable and weighs 200 g. The bandpass is in the range from 1.4–5 nm with a spectral range in the visible to the near-infrared. Henriksen et al. (23) developed a new optomechanical design based on the HSI using commercial off-the-shelf components, which has a spectral resolution of 3.69 nm. The device is also compact, measuring $220\times 65\times 65\;mm^{3}$ and weighing 650 g. Xue et al. (24) developed a highly compact HSI with automatic geometric rectification. The spectral range is from 400 nm to 1,000 nm with an excellent spectral resolution of 2.5 nm. The use of portable HSI and spectrometers contributes to ease and convenient data acquisition for online or in-situ applications, due to the low cost, simplicity of analysis, reduced size, and portability.

Most of spectrometer work independently based on their own spectral range, resolution and experimental conditions (25). Additionally, due to the differences in system configuration, spectral response characteristics and other factors, the data collected on different devices may have systematic bias and error, which affects the generalization ability of the model (26). Repetitive model calibration work makes the application of portable instruments in practical production and market supervision a major obstacle. Model transfer refers to the application of calibration models built on one instrument to other instruments (27). This approach reduces redundant calibration work, improves the portability and robustness of the spectral device and model, and makes the HSI more suitable for in-situ applications. Therefore, systematic research on model transfer and optimization is crucial to improve the performance of meat adulteration detection (28).

In this study, a portable HSI controlled by a Raspberry Pi was designed and optimized for on-site meat quality detection. To address the problem of model incompatibility caused by spectral variations across different instruments, this study evaluated three model transfer methods to enable model sharing between the developed HSI and a commercial spectrometer. Additionally, partial least squares discriminant analysis (PLS-DA) and SVM classifiers were employed to build a meat adulteration discrimination model to visualize the distribution of meat adulteration. This study provides a powerful tool and methodological framework for real-time meat adulteration detection and food safety assurance.

## 2 Materials and methods

**Figure 1:**
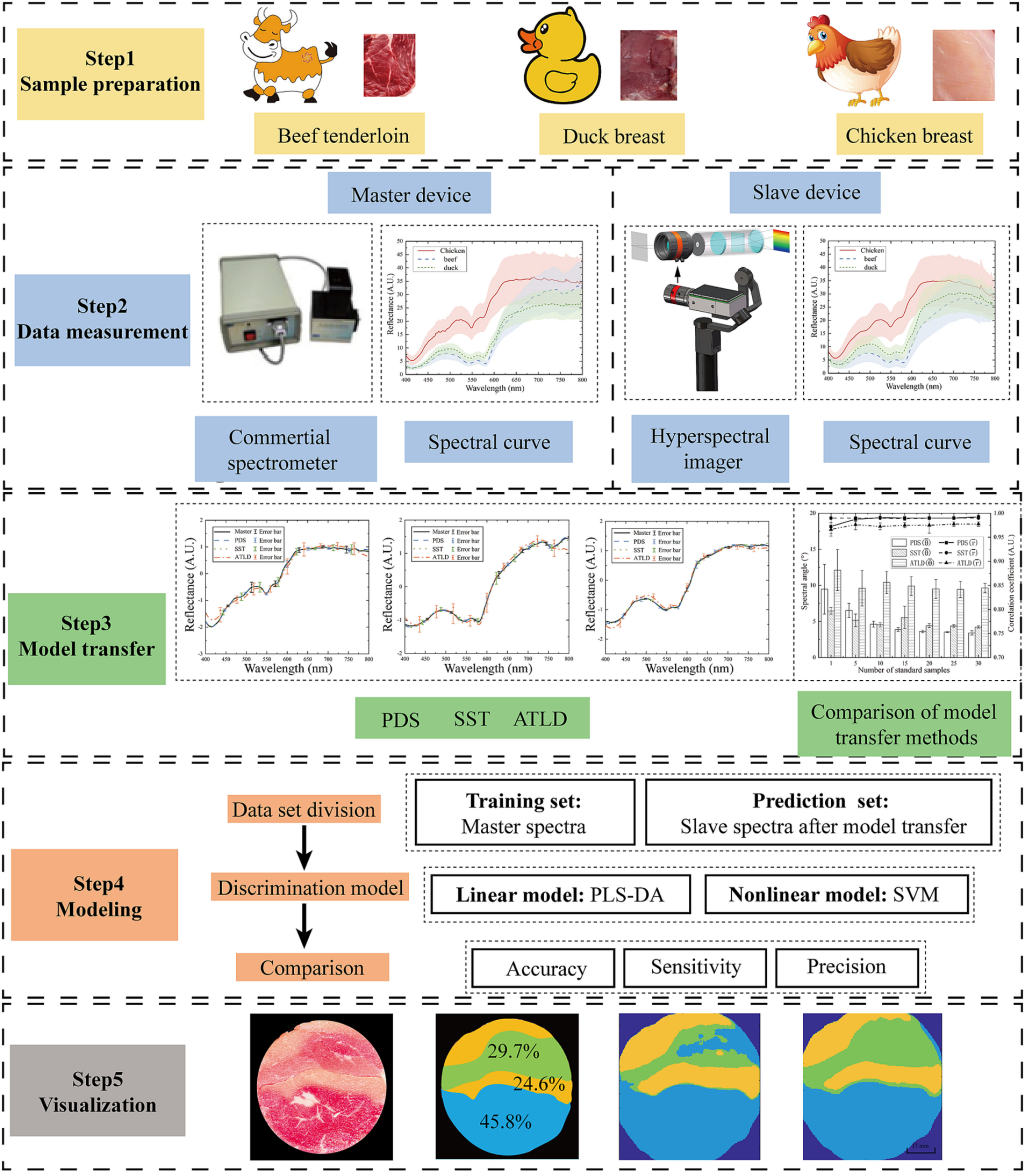
The workflow of meat adulteration discrimination.

This study aims to develop a portable HSI and a discrimination model for in-situ meat adulteration detection. The workflow is illustrated in Figure 1.

The hand-held portable HSI is designed based on the push-scan principle, with Raspberry Pi 4b and custom software to acquire and process the HSI data. The PLS-DA and SVM were employed to establish meat adulteration discrimination model. To enhance model compatibility across different instruments, model transfer methods, including piecewise direct standardization (PDS), SST and alternating trilinear decomposition (ATLD) were evaluated. These model transfer methods were validated on the dataset including a commercial spectrometer and the home-built HSI. Model transfer helps to enable model and data sharing between instruments, improves detection efficiency, and provides a reliable solution for rapid and accurate detection.

### 2.1 Instrument

**Figure 2:**
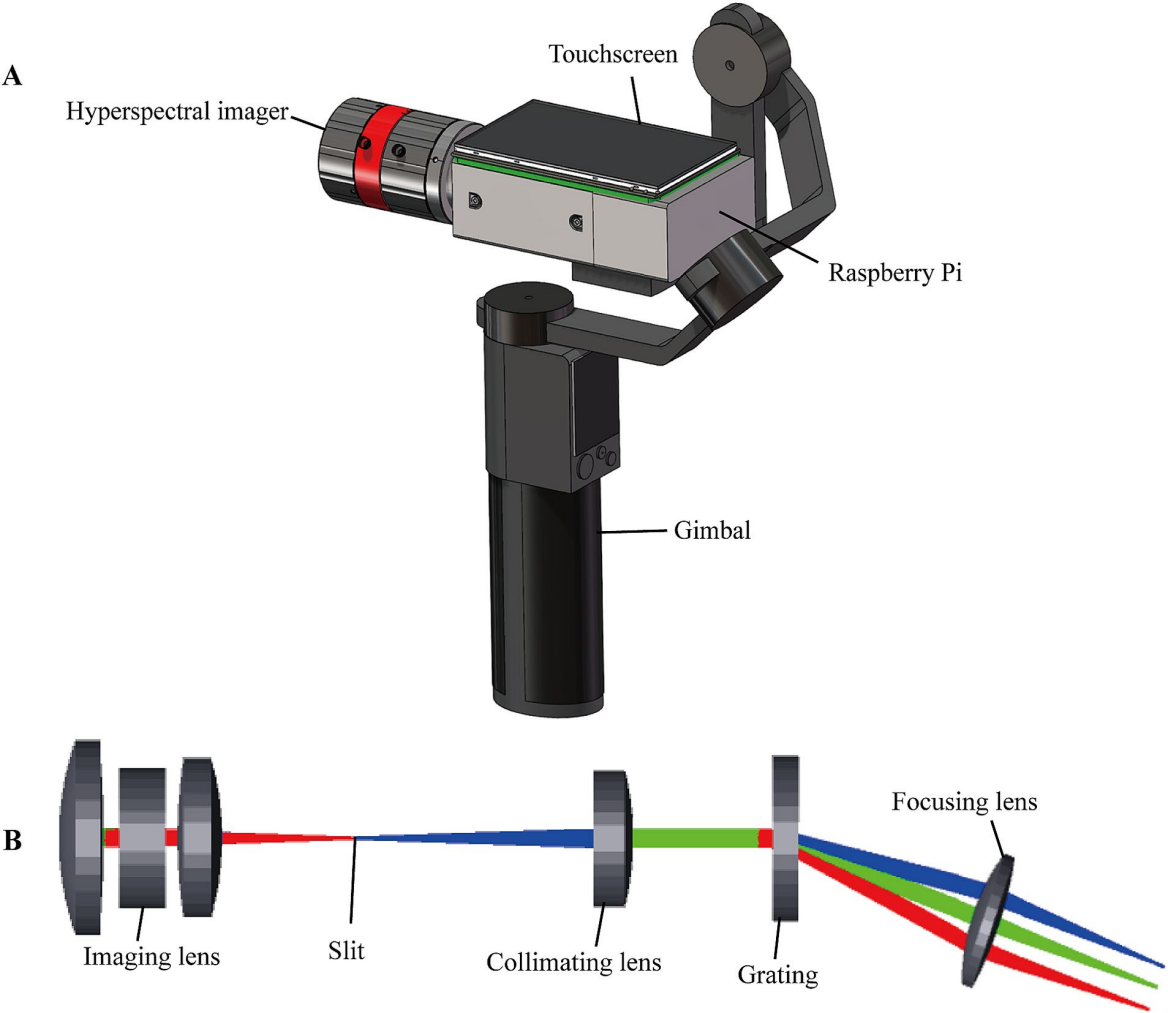
Schematic structure of the portable HSI. **(A)** The portable HSI. **(B)** Physical model of the Zemax simulation.

As shown in Figure 2A, the portable system consists of the push-scan HSI, the Raspberry Pi for real-time image acquisition and processing, a touchscreen and gimbal for user-friendly operation. The HSI covers a spectral range from 400 nm to 800 nm with a center wavelength of 600 nm. To reduce size and cost, the HSI was designed with compact transmission model, which comprises an imaging lens (Focal length, FL = 35 mm), slit (25 μm × 3 mm), collimating lens (FL = 50 mm), grating (600 lines/mm, 25 × 25 mm^2^), focusing lens (FL = 25 mm) and detector (acA2040–120 um, Basler, Germany) for spectrogram acquisition. The optimization in Zemax ray tracing software was performed, as shown in Figure 2B. According to the simulation results, the spectral resolution of the HSI was up to 5 nm with a spatial resolution of 0.1 mm.

### 2.2 HSI calibration

**Figure 3:**
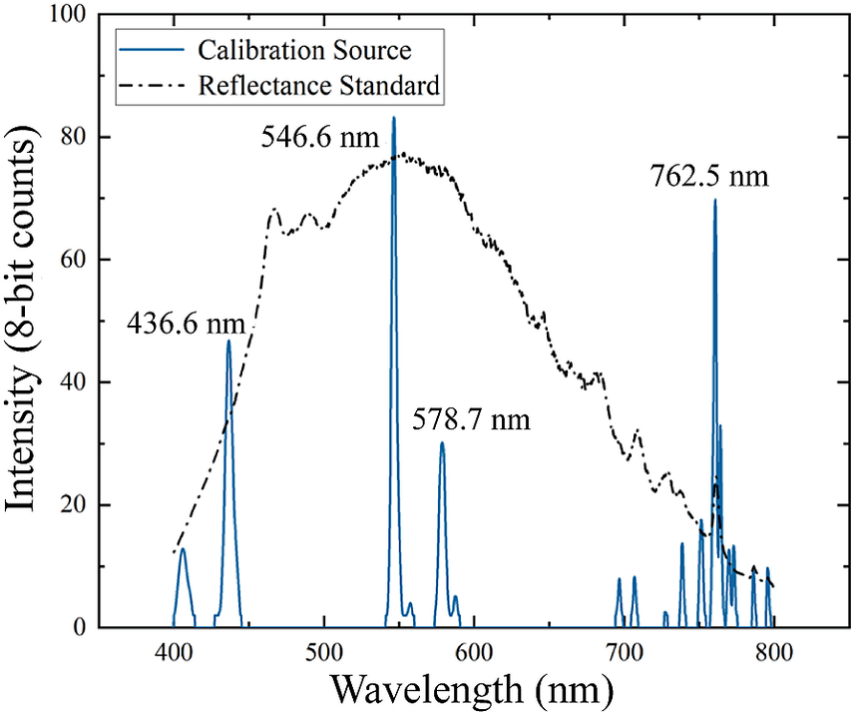
Hyperspectral imager (HSI) calibration data. Blue lines showed spectral calibration peaks from the mercury-argon lamp. The black line was the measured spectrum of the reference standard with Xenon light source.

To evaluate the performance of the HSI, the spectral response and full width at half maxima (FWHM) were measured using a monochromator (CS260B-2-MC-A, Newport, America) and mercury-argon lamp (HG-1, Ocean Optics, America). The monochromator emitted monochromatic light in 1 nm increments across a wavelength range of 400 nm to 800 nm. The light was collected by the HSI, and the response of pixels to different wavelengths was obtained. The relationship between pixels and wavelengths was fitted using the least squares method, and the function is presented as follows (Equation 1):


(1) $$\lambda_{i}=\{\begin{array}{c} -7.44e^{-6}i^{2}-0.201i+796.19\;0<i\leq 944\\ -3.39e^{-6}i^{2}-0.208i+799.5\;944<i\leq 1870 \end{array}$$

where i is the pixel values, $\lambda_{i}$ is the central wavelength of i . The spectral resolution of the HSI was measured using the mercury-argon calibration light source. The FWHM derived from the spectral calibration data in Figure 3, was calculated to be 3.6 nm at 546.6 nm and 3.9 nm at 436.6 nm, which meet the requirement of meat detection.

### 2.3 Sample preparation and experiment

Meat species adulteration is a prevalent issue in the food industry, with a common practice being the partial or complete substitution of expensive beef with cheaper chicken or duck. This substitution can significantly reduce costs while maintaining a similar appearance and flavor in processed meat products. Chicken and duck, with their relatively mild flavors, are particularly difficult to detect when mixed with beef. Meat is also perishable, requiring proper preservation methods to retain its optimum quality until used. Freezing is one of the most effective and efficient methods commonly used in the meat industry to extend the shelf life of meat products (29). However, freezing has also been found to cause a series of physical and biochemical changes in muscle foods, including the formation of ice crystals, solute concentration, changes in ionic strength and pH, freezer burn, discoloration, lipid oxidation and protein denaturation (30).

To investigate the adulteration of frozen meat, fresh beef tenderloin, chicken breast and duck breast were purchased from local supermarkets. One hundred forty-four samples were cut into uniform pieces measuring 3 cm × 3 cm × 0.5 cm and then frozen at −18°C for 24 h. The spliced samples were made with beef, chicken and duck in the proportion of 0.5:1:2, 1:1:1 and 2:1:1, wrapped in cling film and also frozen at −18°C for 24 h, and then cut to uniform size. Since the meat is spliced, the actual proportions of each piece of meat differ from the spliced proportions. The spectral and hyperspectral data of the samples were detected simultaneously using the commercial spectrometer (BTC611E, B&W Tek, United States) and portable HSI, ensuring that each sample was detected within 5 min. The spectrometer was defined as the master instrument in the model transfer method. Its spectral range is 300 nm to 800 nm with the spectral resolution of 1.3 nm. The portable HSI was the slave, with the spectral range of 400 nm to 800 nm, and the spectral resolution of 5 nm.

### 2.4 Model transfer methods

Model transfer through standardization of spectral responses is the most commonly used strategy. These methods find a transfer matrix that standardizes the spectra measured on slave instrument into the feature space of the master spectra (31). It can be directly applied to the prediction model built on the master spectra without significantly reducing the prediction accuracy. Commonly used model transfer methods include direct standardization (DS) and piecewise direct standardization (PDS). The DS employs PLSR to model the multiple regression between the master and slave spectra, generating a transfer matrix for spectral normalization. However, DS performs poorly in handling non-linear differences between devices. To address this limitation, PDS introduces a moving window to estimate the transfer matrix locally, thereby correcting for the non-linear deviation (32). However, PDS is highly sensitive to the window size, and determining the optimal window size becomes challenging, particularly when the number of spectra from the slave instrument is limited. SST constructs the data matrix by aligning spectra measured from different instruments and estimates the transfer matrix based on principal component analysis (PCA) loadings (33). The SST features a relatively simple structure and maintains robust even with a small number of standard samples. In contrast, ATLD is a higher-order standardization method based on 3D data decomposition. ATLD is capable of combining spectral, spatial and sample dimensions in the standardization process and is suitable for modeling and alignment of complex data with high adaptability and robustness (34).

The PDS, SST and ATLD methods were selected in this study to achieve model transfer between the commercial spectrometer and HSI. PDS assumes that the response at a specific wavelength from the master spectra is correlated with the response from the slave within a predefined window. The transfer function is expressed as (Equation 2):


(2) $$S_{1,i}=S_{2,i-w\ldots i+w}B_{i}$$

where the window width is 2w+1 , $S_{1,i}$ is the value of the i wavelength in the master spectra, $S_{2,i-w\ldots i+w}$ is the value within the wavelength range from i–w to i+w in the slave spectra, and $B_{i}$ is the transfer coefficient of the i wavelength. The transfer coefficient B was calculated using PLSR, with the window size optimized based on the RMSE between the master and slave spectra. The performance of PLSR is influenced by the number of principal components. The optimal number was determined using leave-one-out cross-validation, yielding a window size of 25 and 6 principal components for the PDS calibration.

Spectral space transformation integrates the standard sets collected by the master $X_{m}$ and slave $X_{s}$ to create a spectral array $X_{\mathrm{comb}}=\left[X_{m},X_{s}\right]$ . The PCA is then applied to $X_{\mathrm{comb}}$ to obtain the load using the following formula (Equation 3):


(3) $$X_{\mathrm{comb}}=T\left[P_{m}^{T},P_{s}^{T}\right]+E$$

where $P_{m}^{T}$ and $P_{s}^{T}$ are the loads of the master and slave spectra, respectively. The loads are used to calculate the transformation matrix F using the following formula (Equation 4):


(4) $$F=I+P_{s}^{T}+\left(P_{m}^{T}-P_{s}^{T}\right)$$

where I is the unit matrix. Convert the slave spectra to master spectra using the following formula (Equation 5):


(5) $$x_{un}^{p}=x_{un}F$$

Alternating trilinear decomposition is an algorithm for decomposing three-dimensional data arrays. The spectra of standard samples collected from different instruments can be represented as a three-dimensional matrix X with dimensions I×J×K . This matrix can be decomposed using the ATLD algorithm shown as the following equation (Equation 6):


(6) $$x_{ijk}=\overset{N}{\underset{n=1}{\sum }}a_{\mathrm{in}}b_{jn}c_{kn}+e_{ijk}$$

where i,j,k is the number of standard samples, wavelengths and instruments, respectively, N is the number of factors, $a_{\mathrm{in}}$ is the element $\left(i,n\right)$ of an I×N matrix A, $b_{jn}$ is the element $\left(j,n\right)$ of an J×N matrix B, and $c_{kn}$ is the element $\left(k,n\right)$ of an K×N matrix C. The transformation matrix F can be obtained using the following formula (Equation 7):


(7) $$F_{k}=\mathrm{diag}\left(c_{k}\right)B^{T}$$

where $c_{k}$ is the kth row of matrix C . For the spectrum $x_{k1,\mathrm{new}}$ taken from the K1 instrument, it can be transformed into the spectrum $x_{k2,\mathrm{trans}}$ from the K2 instrument by the following equation (Equation 8):


(8) $$x_{ik2,\mathrm{trans}}=x_{ik1,\mathrm{new}}+x_{ik1,\mathrm{new}}F_{k1}^{+}F_{k2}-x_{ik1,\mathrm{new}}F_{k1}^{+}F_{k1}$$

The performance of SST and ATLD is influenced by the number of principal components and factors, respectively. Optimal parameters were identified using five-fold cross-validation, where the RMSE between master and slave spectra was calculated as an evaluation criterion. Ultimately, the number of principal components and factors that minimized the RMSE were chosen to be 6 and 2, respectively.

### 2.5 Discrimination models

Combined with the model transfer method, the meat adulteration discrimination model was developed based on PLS-DA and SVM. The PLS-DA extracts the principal components between the spectral matrix and the categorical variables for linear discrimination, and can effectively deal with the multicollinearity in high-dimensional data (35). The SVM maps data into a high-dimensional feature space for non-linear discrimination. It captures complex non-linear relationships in spectral responses, demonstrates high accuracy in small-sample learning, and exhibits strong generalization ability (36).

The PLS-DA maximizes the covariance between the spectral matrix X and the predicted category Y by selecting the principal components with the following formula (Equation 9):


(9) Y=Xb+e

where b is the matrix of regression coefficients and e is the matrix of residual information. Selecting the appropriate number of principal components is crucial when building the PLS-DA model. Using too many components can result in overfitting, while using too few may lead to the loss of important information. In this study, the optimal number is determined using the 10-fold cross-validation method, with an error threshold of 0.5 for category discrimination.

Support vector machine is a non-linear method with high generalization ability. It maps the spectral vector $x_{i}$ to the high-dimensional feature space using a kernel function. This allows SVM to find the optimal linear separating hyperplane $\left(w,b\right)$ . The main objective is to optimize the following (Equations 10, 11):


(10) $$\begin{array}{c} \underset{w,b}{\min\;}\frac{1}{2}\|w\|{}^{2}\\ s.t.y_{i}\left(w^{T}\phi \left(x_{i}\right)+b\right)\geq 1,i=1,2,\ldots ,m \end{array}$$

(11) $$K\left(x_{i},x_{j}\right)\text{≡}\phi {\left(x_{i}\right)}^{T}\phi \left(x_{j}\right)$$

where $K\left(x_{i},x_{j}\right)$ is the kernel function. The radial basis function is employed as the kernel function, and the optimal kernel parameters are determined through cross-validation.

### 2.6 Model evaluation

Model transfer method requires standard datasets to obtain the transformation matrix, while the discriminative model also requires training dataset. The sample set partitioning based on joint X-Y distances (SPXY) method was used to divide the dataset into training and prediction sets (37). Subsequently, a subset of samples from the training set was selected as the standard set. The number of samples in standard sets was determined by calculating the spectral angles $\bar{\theta }$ (Equation (12)) and correlation coefficients $\bar{r}$ (Equation (13)) (38).


(12) $$\bar{\theta }=\frac{1}{m}\overset{m}{\underset{j=1}{\sum }}\theta_{i},\;\bar{\theta }\in \left[0,\frac{\pi }{2}\right]$$

(13) $$\theta_{i}=\arccos\frac{\sum_{j=1}^{n}M_{ij}S_{ij}}{\sqrt{\sum_{j=1}^{n}M_{ij}^{2}}\sqrt{\sum_{j=1}^{n}S_{ij}^{2}}},\theta \in \left[0,\frac{\pi }{2}\right]$$

where $\theta_{i}$ is the spectral angle between the master and slave spectra of the ith sample, and m is the number of samples in the standard set. $M_{ij}$ and $S_{ij}$ denote the spectral response of the master spectrum of the ith sample and the corresponding jth wavelength in the slave spectrum, respectively. n is the number of wavelengths in the spectral curve. The mean value of the spectral correlation coefficient can be expressed as (Equations 14, 15):


(14) $$\bar{r}=\frac{1}{m}\overset{m}{\underset{i=1}{\sum }}r_{i},\;\bar{r}\in \left[0,1\right]$$

(15) $$r_{i}=\frac{\sum_{j=1}^{n}\left(M_{ij}-\bar{M_{i}}\right)\left(S_{ij}-\bar{S_{i}}\right)}{\sqrt{\sum_{j=1}^{n}{\left(M_{ij}-\bar{M_{i}}\right)}^{2}}\sqrt{\sum_{j=1}^{n}{\left(S_{ij}-\bar{S_{i}}\right)}^{2}}},r\in \left[0,1\right]$$

As the value of $\bar{r}$ approaches 1 and $\bar{\theta }$ approaches 0, the difference between the master and slave spectra decreases, indicating a more effective model transfer method. The performance of discriminant models is typically evaluated using confusion matrix, in which four commonly used evaluation metrics can be calculated: accuracy, sensitivity, and precision (39). The formulas for these metrics are as follows (Equations 16–18):


(16) $$\mathrm{Accuracy}=\frac{TP+TN}{TP+TN+FP+FN}$$

(17) $$\mathrm{Sensitivity}=\frac{TP}{TP+FN}$$

(18) $$\mathrm{Precision}=\frac{TP}{TP+FP}$$

## 3 Results and discussion

### 3.1 Spectral analysis

**Figure 4:**
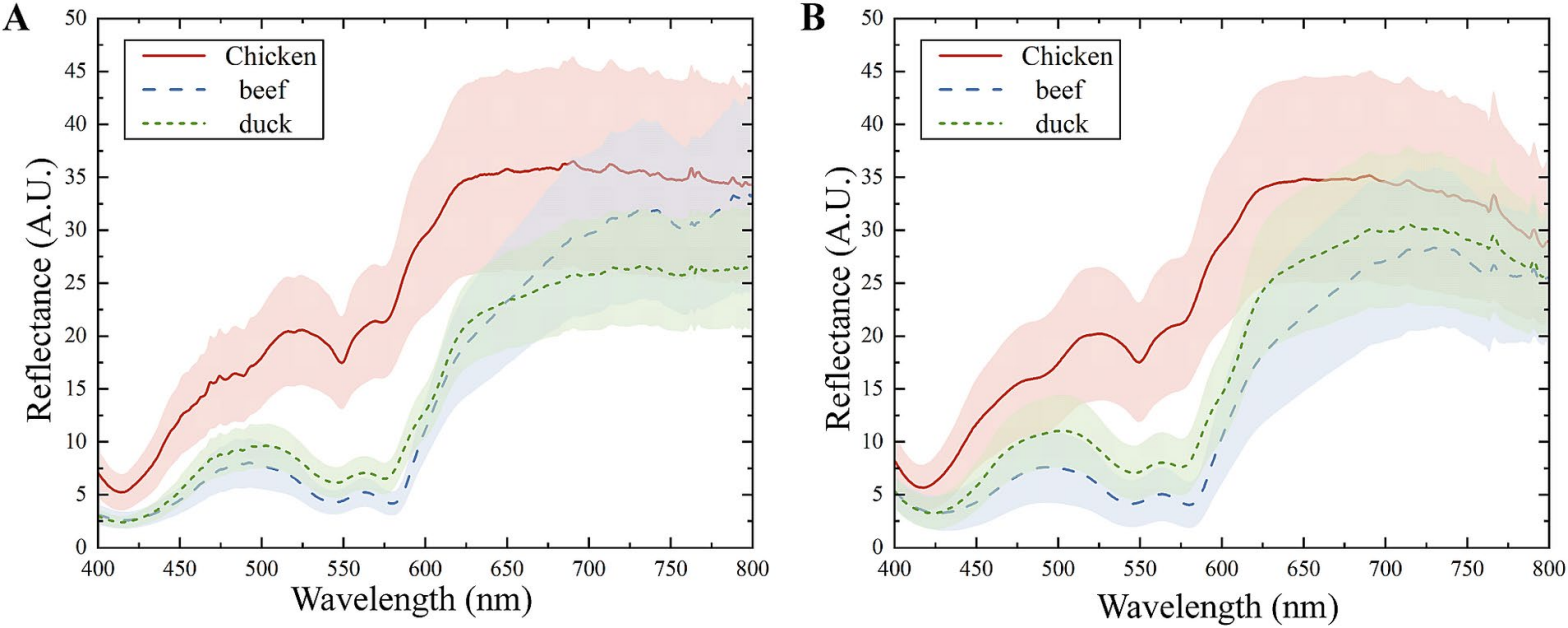
The reflectance spectra of meat measured by the master and slave. **(A)** The commercial spectrometer. **(B)** The HSI.

As shown in Figure 4, the reflectance spectra of three types of meat shows different intensity and absorption features in the range of 400–800 nm. The absorption peaks at 420 nm, 540 nm and 575 nm differ significantly, primarily due to variations in myoglobin content (40). The peak at 420 nm corresponds to deoxymyoglobin, while the peaks at 540 nm and 575 nm are associated with respiratory pigments, such as oxymyoglobin (41). The content and distribution of myoglobin influence both the spectral intensity and meat color. The spectra of beef and duck are similar, whereas chicken exhibits higher spectral reflectance, likely due to differences in respiratory pigment composition. Furthermore, a minor absorption peak at 630 nm is observed in the beef spectrum, attributed to the presence of high-iron myoglobin (42).

**Figure 5:**
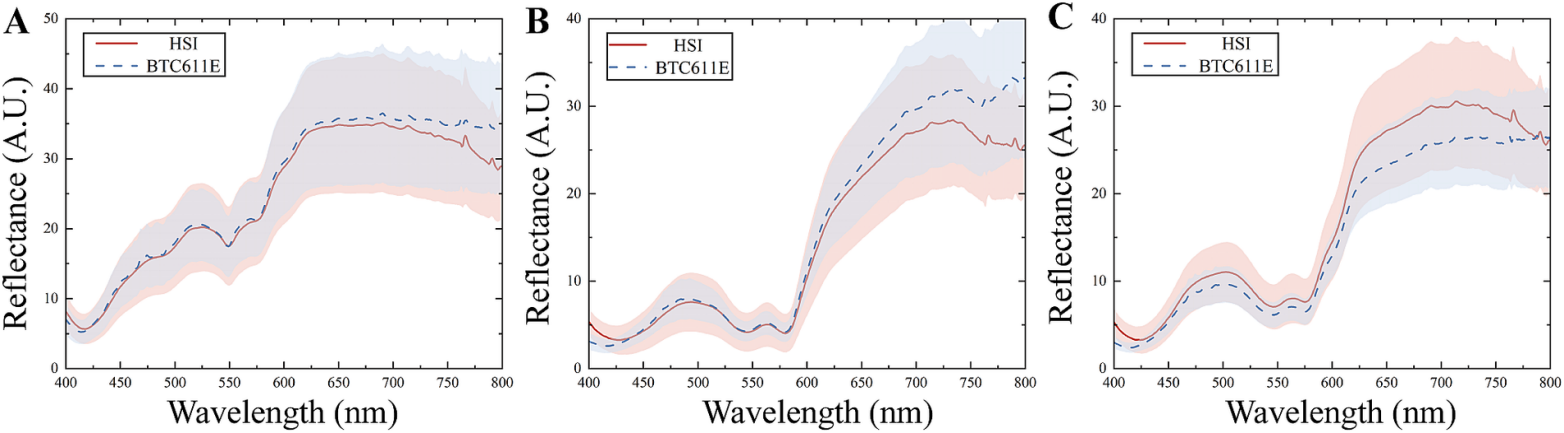
Reflectance spectra of meat measured with the HSI and commercial spectrometer. **(A)** Chicken; **(B)** beef; **(C)** duck.

The reflectance spectra of meat measured with the HSI and commercial spectrometer are compared in Figure 5. Differences between the master and slave spectra are attributed to instrument design, measurement conditions and other factors. The discrepancies are particularly pronounced in the spectral ranges of 400–425 nm and 600–650 nm. Applying the discrimination model developed on the master to spectra measured from the slave could lead to significant prediction errors. Therefore, it is necessary to minimize the discrepancies between the master and slave and improve the discrimination accuracy through model transfer methods.

### 3.2 Comparison of model transfer methods

**Figure 6:**
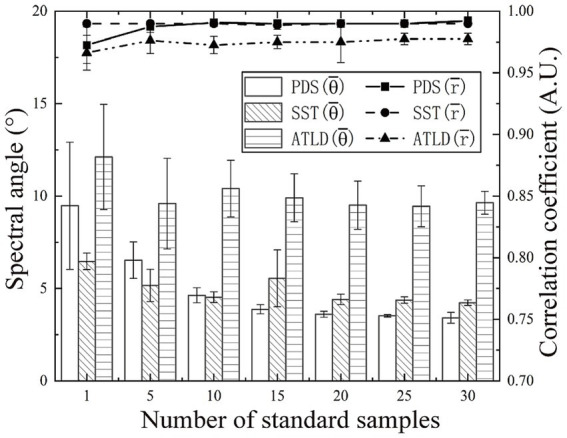
Impact of the number of standard samples on the performance of the model transfer approach.

In order to maintain the prediction performance of the discrimination models, three model transfer methods (PDS, SST, and ATLD) were used to minimize the effects of instrumental variations, and their performance was evaluated and compared. The number of standard samples has a significant effect on the performance of model transfer methods. In general, the greater the number of standard samples, the higher the possibility of obtaining good results. However, in practice, model transfer methods that can obtain satisfactory results using fewer standard samples are preferred because fewer standard samples require less analysis time and lower costs. As shown in Figure 6, there is an overall decreasing trend in the spectral angle between the master and slave spectra as the number of standard samples increases. However, after reaching a certain threshold, further increases the number of standard samples have little increase in the spectral angle, as excessive samples tend to introduce redundant information and noise. Specifically, when the number of standard samples ranges from 1 and 10, the SST method achieved the smallest spectral angle, indicating that SST can provide a better standardization at a lower number of standard samples. The spectral angle for the PDS method is minimized when the number of standard samples falls within the range of 15 to 30. In contrast, the ATLD method consistently exhibits a larger spectral angle, indicating a significant difference between the master and transformed slave spectra. Based on these results, 15, 10, and 20 standard samples were selected for the PDS, SST, and ATLD, respectively.

**Figure 7:**
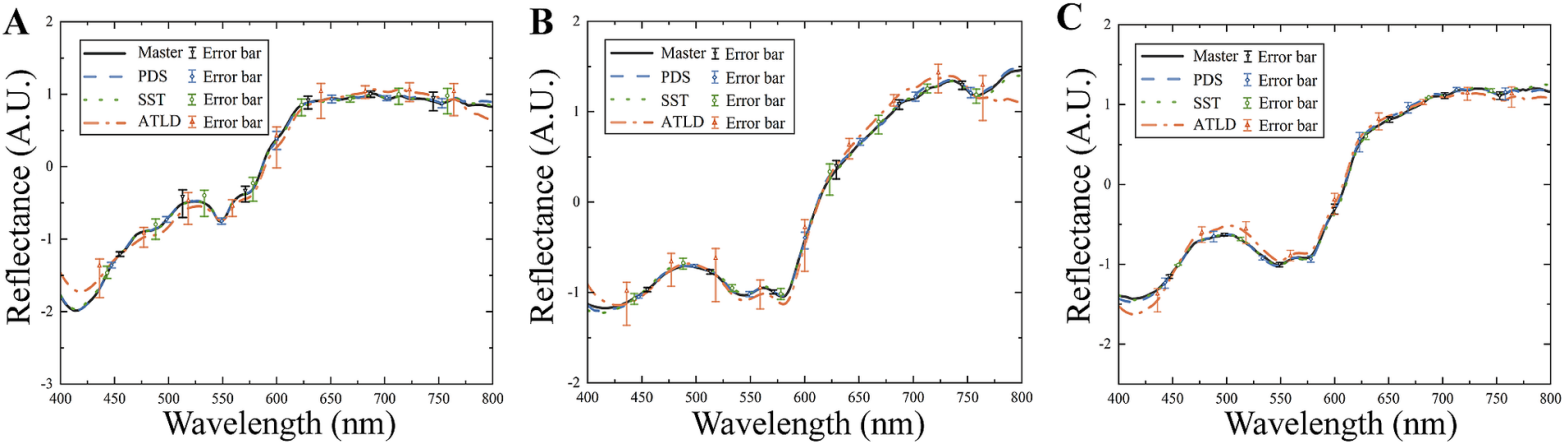
Comparison of master and slave spectra before and after model transfer. **(A)** Chicken; **(B)** beef; **(C)** duck.

The spectral angles and correlation coefficients between the master and slave spectra before and after the model transfer are compared in Table 1. After model transfer, the spectral angles were significantly reduced. Specifically, the PDS method has the best performance with the spectral angle reduced to 27% of the value before transfer, while the SST method has the spectral angle reduced to 31% of the original one. As shown in Figure 7, the transformed slave spectra exhibit similar shape with the master spectra, further confirming the effectiveness of these methods in correcting spectral variations caused by changes in instrumental or experimental conditions.

**Table 1:** The spectral angles and correlation coefficients between master and slave spectra before and after model transfer.

Methods	Number of standard samples	Spectral angle (°)	Correlation coefficient
No	0	14.53	0.90
PDS	15	3.87	0.99
SST	10	4.53	0.99
ATLD	20	9.50	0.97

SST, spectral space transformation; PDS, piecewise direct standardization; ATLD, alternating trilinear decomposition.

### 3.3 Comparison of discrimination results before and after model transfer

The effects of different model transfer methods on discrimination accuracy are compared in Table 2. Before transfer, applying the discrimination model developed on the master to the slave lead to low accuracy, with PLS-DA and SVM achieving 82.63 and 90.51%, respectively. Model transfer significantly improves accuracy of classifiers, with the SST method increasing accuracy by 9.73% for PLS-DA and 4.4% for SVM. The PDS method improves accuracy by 7.18% for PLS-DA and 1.62% for SVM. The ATLD method improves classification accuracy, with PLS-DA and SVM increasing by 6.72 and 1.08%, respectively. However, its improvement is limited compared to PDS and SST. The ATLD method employs trilinear decomposition to separate spectra into feature matrices related to category, spectrum and instrument. It iteratively minimizes the squared error between the original and reconstructed data, making the calculation complex (34). Additionally, the ATLD method calculates the transfer matrix using only the feature matrices associated with the spectra and instrument, potentially omitting critical information that can affect model transfer and classification accuracy (43).

**Table 2:** The discrimination accuracy of slave test set before and after model transfer.

Model transfer	Methods	Discrimination accuracy (%)	
		PLS-DA	SVM
No	No	82.63	90.51
Yes	PDS	89.81	92.13
	SST	92.36	94.91
	ATLD	89.35	91.59

SST, spectral space transformation; PDS, piecewise direct standardization; ATLD, alternating trilinear decomposition; PLS-DA, partial least squares discriminant analysis; SVM, support vector machine.

**Figure 8:**
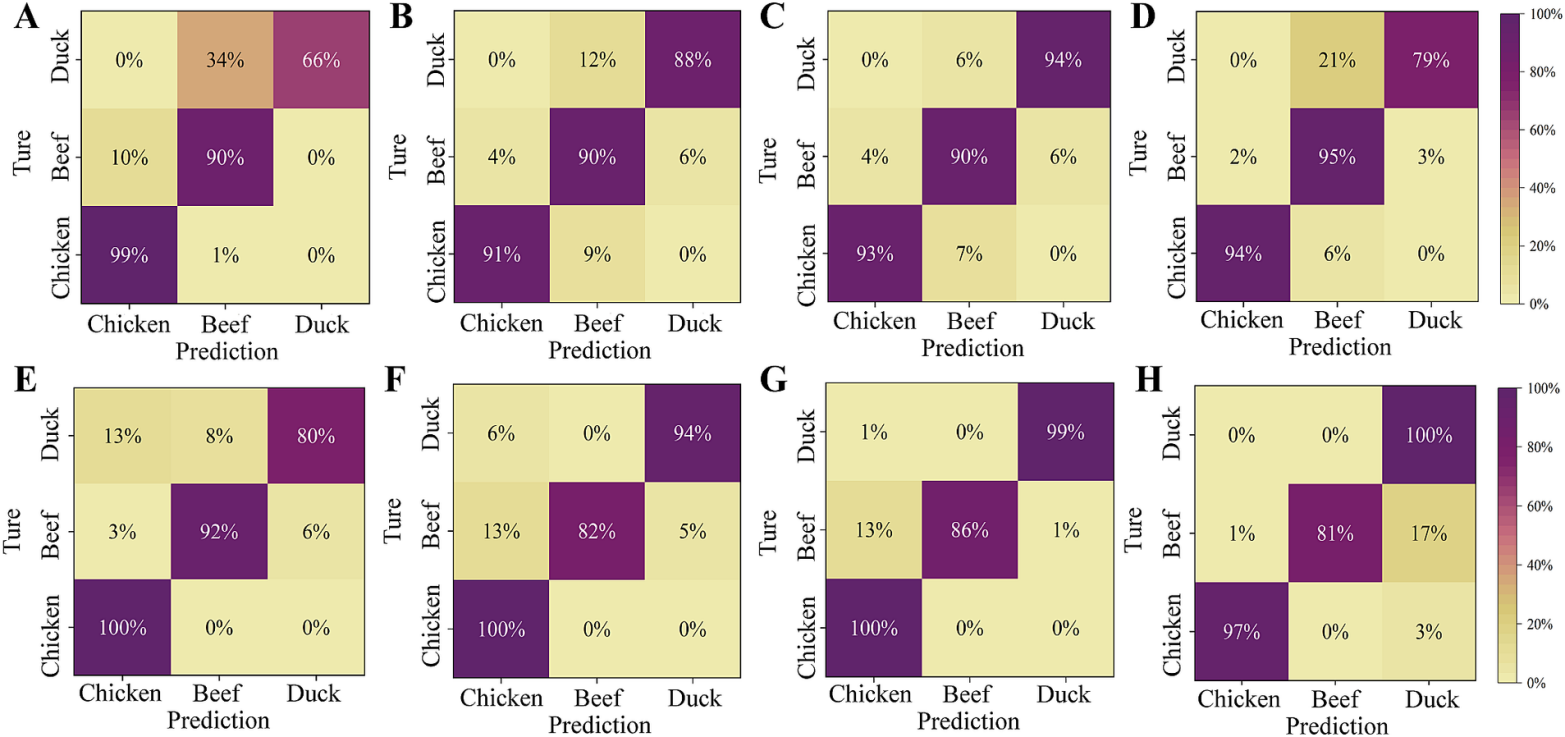
Confusion matrix for the slave test set before and after model transfer. **(A)** PLS-DA model (before transfer); **(B)** PLS-DA model based on PDS; **(C)** PLS-DA model based on SST; **(D)** PLS-DA model based on ATLD; **(E)** SVM model (before transfer); **(F)** SVM model based on PDS; **(G)** SVM model based on SST; **(H)** SVM model based on the ATLD.

In the task of multi-class classification, there inevitably exists differences among the classification abilities of a classifier to different classes (44). Such differences are hard to be reflected by any single performance index. Confusion matrices contain information about the actual and predicted classifications given by a classifier. Based on the data from the confusion matrix, accuracy, sensitivity, specificity, and precision can be calculated and used to evaluate the performance of the model (39). As shown in Figure 8, the PLS-DA and SVM classifiers had the best discrimination on chicken with a sensitivity of over 99% before model transfer. However, the sensitivities for beef and duck are lower. This is because chicken exhibits higher spectral reflectance due to its high deoxymyoglobin content, resulting in a light yellow or purplish red color. In contrast, beef and duck exhibit bright red colors with similar spectral characteristics, and spectral variation due to different instruments further increased their misclassification rates. The sensitivity for duck is significantly improved after the model transfer. Specifically, the sensitivity for duck in Figures 8C,G increases by 28 and 19%, respectively. However, Figures 8F–H shows that the sensitivity of SVM for beef discrimination decreases. This may be due to partial overlap between categories in the feature space after model transfer, and improving the accuracy for one category may reduce performance of others.

### 3.4 Visualization of meat adulteration

**Figure 9:**
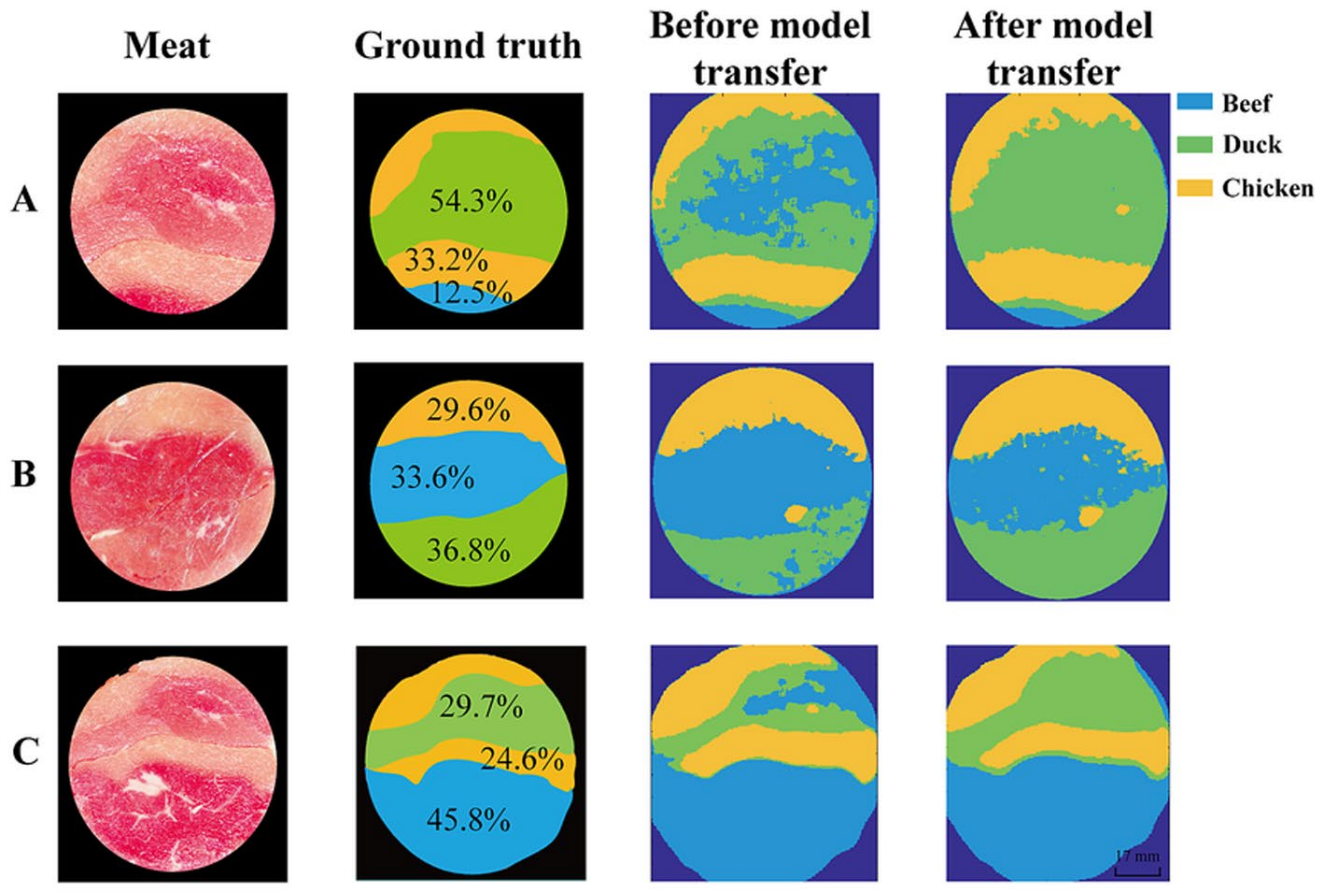
Visualization maps of meat adulteration. Proportion of beef, chicken and duck in spliced meat is **(A)** 0.5:1:2; **(B)** 1:1:1; **(C)** 2:1:1. Chicken–yellow, beef– blue, and duck–green.

Hyperspectral images contain abundant spatial information, and visualization clearly displays the spatial distribution of meat adulteration. This study employed the SVM classifier based on SST to discriminate the hyperspectral images with different proportions of spliced meat. The results are shown in Figure 9, where chicken, beef, and duck regions are represented in yellow, blue, and green, respectively. Since the sample preparation was done by slicing the meat after it was frozen and spliced, the actual proportion of each piece of meat differed from the spliced proportion. The comparison between the actual and predicted proportions of adulteration meat is presented in Table 3.

**Table 3:** Proportion of meat adulteration.

Image no.	Calibration data	Prediction data	Model transfer	Proportion of meat (%)		
				Beef	Duck	Chicken
A	Ground truth	No	No	12.5	54.3	33.2
	Spectrometer	HSI	No	33.5	36.7	29.8
			Yes	10.3	54.1	35.6
B	Ground truth	No	No	33.6	36.8	29.6
	Spectrometer	HSI	No	50.1	24.3	25.6
			Yes	36.8	32.9	30.3
C	Ground truth	No	No	45.7	29.7	24.6
	Spectrometer	HSI	No	52.7	18.8	28.5
			Yes	41.3	33.9	24.8

As shown in Figure 9, before model transfer, the proportion of beef differ from the ground truth by 21.0, 16.5, and 7.0%, respectively. This is primarily due to the misclassification of duck as beef in most of the region, with the proportion of duck differing from the ground truth by 17.6, 12.5 and 10.9%, respectively. After model transfer, the deviations were significantly reduced, with beef proportion differing by only 2.2, 3.2, and 4.4%, while duck proportion deviated by 0.2, 3.9, and 4.2%, respectively. These results demonstrate that the model transfer method can effectively correct the spectral variations caused by different instruments, improve the generalization ability and robustness of the model, and provide a reliable solution for the real-time detection.

In this study, the HSI was utilized to capture the spectral characteristics of different meat species, and a discrimination model was constructed to predict adulteration proportion in spliced meat. The SVM combined with SST method gave optimal results with an accuracy of 94.91% in the meat species discrimination. The discrimination sensitivities for chicken, duck and beef were 100, 86 and 99%, respectively. However, this study did not quantitatively predict the content of adulteration meat. Zhang et al. (16) used RP-CNN to discriminate pork adulteration in mutton, and the discrimination accuracy can reach 100%. The study also established a model for quantitative predicting the pork content in fresh and frozen–thawed meat. The $R^{2}$ on two datasets of fresh and frozen–thawed samples were 0.9762 and 0.9807, respectively. Jiang et al. (45) employed hyperspectral imaging to detect the offal adulteration in ground pork. The best performance of the PLSR model was achieved with the $R^{2}$ of 0.98 and the RMSE of 4.47%. However, the samples were all purchased from a supermarket in the same day, which may limit the applicability of the model to a wider range of scenarios. In many studies, samples have been prepared by mixing different proportions of the adulteration meat. New samples and models have to be prepared to cope with complex forms of adulteration, making the process time-consuming, costly, and limiting the models’ applicability and generalization.

The portable HSI enables on-site analysis, making it an invaluable tool for various industries, including food safety and quality control. However, the application of portable HSI in practice still faces several challenges. Variations in ambient lighting conditions and surface contaminants on meat samples—such as moisture and grease—can modify the spectral and imaging characteristics, compromising the accuracy of instrument (14). Future research should focus on optimizing calibration algorithms and instrument design. Additionally, integrating deep learning with hyperspectral imaging to develop end-to-end models can streamline preprocessing, automate feature extraction, and enhance the efficiency of real-time detection (46).

## 4 Conclusion

In this study, a portable HSI and a discrimination model were proposed for detecting chicken and duck adulteration in beef. The HSI is controlled by the Raspberry Pi with the spectral resolution of 5 nm and spatial resolution of 0.1 mm. With model transfer, the slave spectra were normalized to the feature space of the master spectra and used further as input of classifiers. The results demonstrated that the model transfer method effectively reduces the spectral differences due to instrumental variations, and the spectral angle reduced to 31% of the value before transfer with SST. The performance of the SVM classifier was greatly improved with SST, achieving a prediction accuracy of above 94.91%. The discrimination sensitivities for chicken, duck and beef were 100, 86 and 99%, respectively. The study indicating the great potential of the hyperspectral technology applying in the meat adulteration not only the duck and chicken adulteration in spliced beef. Recently, an increasing number of portable instruments have been developed to meet the growing demand for rapid food analysis. This study attempts to apply models developed on commercial spectrometers to home-built HSI, offering the possibility of integrating portable spectrometers into digital supply chains in the future. This will help to achieve transparency and traceability of the food transport process to ensure life and health safety.

## Data Availability

The raw data supporting the conclusions of this article will be made available by the authors, without undue reservation.
